# A comparative analysis of imaging-based algorithms for detecting focal cortical dysplasia type II in children

**DOI:** 10.1038/s41598-025-16015-3

**Published:** 2025-08-15

**Authors:** Jan Šanda, Zuzana Holubová, David Kala, Kateřina Jiránková, Martin Kudr, Tomáš Masák, Anežka Bělohlávková, Pavel Kršek, Jakub Otáhal, Martin Kynčl

**Affiliations:** 1 Department of Radiology, Second Faculty of Medicine, Charles University and Motol University Hospital, Prague, Czech Republic; 2 Department of Pathophysiology, Second Faculty of Medicine, Charles University, Prague, Czech Republic; 3 Department of Pediatric Neurology, Second Faculty of Medicine, Charles University and Motol University Hospital, Prague, Czech Republic; 4 Epilepsy Research Center Prague, EpiReC, Prague, Czech Republic; 5 Institute for Statistics and Mathematics, WU Vienna, Vienna, Austria; 6 European Reference Network EpiCARE, Prague, Czech Republic

**Keywords:** Focal cortical dysplasia type II, Pediatric epilepsy, Cortical thickness, Gray-white matter blurring, Neuroimaging, Epilepsy, Magnetic resonance imaging

## Abstract

Focal cortical dysplasia (FCD) is the leading cause of drug-resistant epilepsy (DRE) in pediatric patients. Accurate detection of FCDs is crucial for successful surgical outcomes, yet remains challenging due to frequently subtle MRI findings, especially in children, whose brain morphology undergoes significant developmental changes. Automated detection algorithms have the potential to improve diagnostic precision, particularly in cases, where standard visual assessment fails. This study aimed to evaluate the performance of automated algorithms in detecting FCD type II in pediatric patients and to examine the impact of adult versus pediatric templates on detection accuracy. MRI data from 23 surgical pediatric patients with histologically confirmed FCD type II were retrospectively analyzed. Three imaging-based detection algorithms were applied to T1-weighted images, each targeting key structural features: cortical thickness, gray matter intensity (extension), and gray–white matter junction blurring. Their performance was assessed using adult and pediatric healthy controls templates, with validation against both predictive radiological ROIs (PRR) and post-resection cavities (PRC). The junction algorithm achieved the highest median dice score (0.028, IQR 0.038, *p* < 0.01 when compared with other algorithms) and detected relevant clusters even in MRI-negative cases. The adult template (median dice score 0.013, IQR 0.027) significantly outperformed the pediatric template (0.0032, IQR 0.023) (*p* < 0.001), highlighting the importance of template consistency. Despite superior performance of the adult template, its use in pediatric populations may introduce bias, as it does not account for age-specific morphological features such as cortical maturation and incomplete myelination. Automated algorithms, especially those targeting junction blurring, enhance FCD detection in pediatric populations. These algorithms may serve as valuable decision-support tools, particularly in settings where neuroradiological expertise is limited.

**Supplementary Information:**

The online version contains supplementary material available at 10.1038/s41598-025-16015-3.

## Introduction

Focal cortical dysplasia (FCD) refers to a spectrum of focal brain malformations characterized by disordered cortical lamination, with or without abnormal cell types^1^. The classification is based on the type of disturbance of the cytoarchitectonics of the cortical layers, potentially in conjunction with the presence of atypically developed and morphologically altered cells, such as dysmorphic neurons (type IIA) and balloon cells (type IIB)^2^. FCDs constitute the most prevalent cause of focal drug-resistant epilepsy (DRE) in children, occurring in more than half of pediatric patients (and one-quarter of adults)^3,4^.

Detection and accurate localization of epileptogenic lesion are crucial for the success of subsequent surgical treatment of patients with DRE types. The fundamental prerequisites are the quality of MR scans in the form of a dedicated MRI protocol for structural imaging^5^ and appropriate professional interpretation of radiological images by a radiologist experienced in the evaluation of specific epileptological findings^6^. Moreover, the use of advanced MRI techniques, has been shown to improve detection rates and characterization of FCD lesions. Although 3 T MRI generally offers superior sensitivity, particularly for identifying subtle lesions, optimized 1.5T MRI protocols can still yield diagnostically useful results in clinical practice^7,8^. FCD type II is more prevalent FCD type with excellent outcomes in a majority of operated patients, however, patients are frequently referred late to epilepsy centers because the dysplastic lesions are commonly overlooked at initial evaluation of MRI scans. Imaging findings of FCD type II encompass focal changes of cortical thickness, abnormal gyral and sulcal pattern, cortical and subcortical signal abnormalities, blurring of the gray-white matter junction, and a radially oriented and funnel-shaped high T2/FLAIR signal intensity in the subcortical white matter pointing to the ipsilateral ventricle (‘transmantle sign’)^1^. Nevertheless, despite dedicated MR imaging and visual interpretation of images by a specialist, approximately 15–30% of findings in patients with focal DRE remain undetected^9^. FCD type I is particularly challenging to detect, with sensitivity rates reported at around 31%^10^. In contrast, FCD type IIb, which is characterized by the presence of balloon cells, shows a much higher detection sensitivity of approximately 90%^10^.

Because of the subtle changes associated with FCD that are often barely detectable on standard MRI, various processing algorithms have been developed to aid in automatic detection. Advanced post-processing techniques, such as voxel-based morphometry (VBM) and surface-based classification (SBC), have demonstrated the ability to enhance the identification of key structural abnormalities, including cortical thickness alterations and gray-white matter junction blurring^11,12^. Similarly, machine learning and deep learning algorithms, such as convolutional neural networks (CNNs) trained on FLAIR sequences, have shown promise in automating lesion segmentation, particularly in MRI-negative cases^13–15^. Despite these advancements, the diagnostic performance of these algorithms remains clinically suboptimal due to concerns regarding reproducibility, generalizability, and reliability across diverse datasets and clinical environments^16^. Small sample sizes and imbalanced datasets often lead to performance variability, and techniques such as data harmonization and regularization have been suggested to improve their robustness^16,17^.

Moreover, while the combination of quantitative neuroimaging and pattern learning methods has improved group-level diagnostic accuracy, challenges remain in validating these algorithms at the individual patient level. This is critical as many surgically confirmed FCD lesions still go undetected even with enhanced imaging resolution and signal-to-noise ratios^18,19^. Studies have further underscored the need for robust prospective evaluations and cross-cohort validations to ensure the algorithms are reliable and broadly applicable in clinical practice^20–22^. While integrated systems for automated detection show potential, the accuracy and consistency of predictions across varied patient populations must be addressed before widespread clinical implementation can be achieved^23^.

The aim of our study was to evaluate the diagnostic accuracy of structural MRI-based algorithms in detecting FCD types IIA and IIB in pediatric patients with drug-resistant epilepsy. This study extends previous work by introducing two key methodological advances. First, we systematically compared algorithm performance using both adult and pediatric normative templates, addressing the often-overlooked impact of age-appropriate controls on detection accuracy. Second, we applied a dual-reference validation strategy by benchmarking algorithm outputs against both predictive radiological regions of interest (PRR), identified by expert neuroradiologists, and resection cavities corresponding to surgical plans defined by a multidisciplinary clinical team. This approach enabled a comprehensive assessment of the algorithms’ clinical relevance. By analyzing the concordance between algorithm-identified regions, manually outlined areas, and final resection outcomes, we sought to directly evaluate the added value of automated approaches in enhancing detection accuracy and optimizing surgical planning.

## Materials and methods

### Subject selection and ethical compliance

This retrospective study included patients who participated in the epilepsy surgery program at the Motol Epilepsy Center, Prague, Czech Republic, between 2012 and 2020. Inclusion criteria encompassed individuals older than one year with focal DRE, surgical resection, histopathological confirmation of FCD type II, and availability of artefact-free high-quality MR scans suitable for analysis. Additional exclusion criteria included incomplete clinical records or insufficient postoperative follow-up data. Healthy control datasets comprising pediatric and adult volunteers were used as a normative database. Subjects in the control groups were free of neurological or psychiatric disorders, and all MRI scans were carefully reviewed to exclude any structural abnormalities.

All patients and control subjects provided informed consent for study participation, data collection, and data presentation prior to inclusion. For minors, informed consent was obtained from a parent and/or legal guardian. The study protocol was approved by the Ethics Committee of the Motol University Hospital (reference number EK-875.1.42/20) and adhered to the principles outlined in the Declaration of Helsinki.

### Characterization of cohort

We systematically reviewed a cohort of 30 pediatric patients who underwent surgery for focal DRE at Motol Epilepsy Center, Prague, between 2012 and 2020. After excluding 7 patients due to secondary pathology or unsuitable data for analysis, the final cohort comprised 23 patients (10 females, 13 males, see Table 3) with a mean age of 12.9 ± 4.1 years (range: 2.5–18.1 years). The reason for removing patients from the cohort was very young age and incomplete myelination due to subsequent data processing (6 patients aged 0.6–2.7 years). One patient was excluded due to the finding of a cystoid mass in the right hippocampus. All patients had histologically confirmed FCD type II, with 3 cases of FCD type IIA and 20 cases of FCD type IIB, classified according to the ILAE classification^24^.

Two control datasets were used as normative references. The pediatric control group consisted of 36 healthy volunteers (21 females, 15 males) with a mean age of 9.2 ± 1.6 years (range: aged 6.3 to 12.3 years). The adult control group included 81 healthy individuals (35 females, 46 males) with a mean age of 27.5 ± 9.5 years (range 14.6 to 54.7 years).

**Table 1:** Characteristics of individual subjects with FCD type II enrolled into the study and MRI parameters 3D T1-weighted sequences./MUH- motol university hospital; JL-JL Clinic,Prague/.

Subject	Histology	Age [Y]	MRI	TR [ms]	TE [ms]	Flip angle	Voxel size [mm]	FOV [mm]	ILAE (two years after resection)
Subj01	2B	17,1	JL (3T)	8.1	3.7	8	1 × 0.5 × 0.5	240 × 240	4
Subj02	2B	12,2	JL (3T)	8.1	3.7	8	1 × 0.5 × 0.5	240 × 240	1
Subj03	2 A	18,0	JL (3T)	8.1	3.7	8	1 × 0.5 × 0.5	240 × 240	1
Subj04	2 A	6,9	FNM (1.5T)	7.2	3.4	8	1.2 × 1 × 1	240 × 240	1
Subj05	2B	13,3	JL (3T)	8.1	3.7	8	1 × 0.5 × 0.5	240 × 240	1
Subj06	2B	14,7	JL (3T)	8.1	3.7	8	1 × 0.5 × 0.5	240 × 240	1
Subj07	2B	12,3	JL (3T)	8.1	3.7	8	1 × 0.5 × 0.5	240 × 240	1
Subj08	2B	12,9	JL (3T)	8.1	3.7	8	1 × 0.5 × 0.5	240 × 240	4
Subj09	2B	16,6	JL (3T)	8.1	3.7	8	1 × 0.5 × 0.5	240 × 240	1
Subj10	2B	9,1	JL (3T)	8.1	3.7	8	1 × 0.5 × 0.5	240 × 240	1
Subj11	2 A	8,0	JL (3T)	8.1	3.7	8	1 × 0.5 × 0.5	240 × 240	1
Subj12	2B	15,5	JL (3T)	8.1	3.7	8	1 × 0.5 × 0.5	240 × 240	1
Subj13	2B	15,3	JL (3T)	8.1	3.7	8	1 × 0.5 × 0.5	240 × 240	1
Subj14	2B	13,0	JL (3T)	8.1	3.7	8	1 × 0.5 × 0.5	240 × 240	1
Subj15	2B	10,1	JL (3T)	8.1	3.7	8	1 × 0.5 × 0.5	240 × 240	1
Subj16	2B	15,6	JL (3T)	8.1	3.7	8	1 × 0.5 × 0.5	240 × 240	1
Subj17	2B	18,1	JL (3T)	8.1	3.7	8	1 × 0.5 × 0.5	240 × 240	1
Subj18	2B	17,5	JL (3T)	8.1	3.7	8	1 × 0.5 × 0.5	240 × 240	1
Subj19	2B	16,7	FNM (1.5T)	25	4.6	30	1 × 0.8 × 0.8	230 × 230	1
Subj20	2B	6,1	FNM (1.5T)	7.3	3.4	8	1 × 1 × 1	256 × 256	1
Subj21	2B	16,0	JL (3T)	8.1	3.7	8	1 × 0.5 × 0.5	240 × 240	1
Subj22	2B	2,5	FNM (1.5T)	7.4	3.5	8	1 × 1 × 1	256 × 256	1
Subj23	2B	11,8	JL (3T)	8.1	3.7	8	1 × 0.5 × 0.5	240 × 240	1

### MRI data collection

Structural T1-weighted MRI brain scans of FCD cohort were collected at 2 participating centres: Department of Radiology of Motol University Hospital (MUH) and JL Clinic (JL) using two scanners: Philips Achieva 1.5T (MUH) and Philips Achieva 3 T (JL) (Philips Healthcare, Best, the Netherlands). MRI examinations were performed using advanced epilepsy imaging protocols consistent with current HARNESS-MRI recommendations^2^. For radiological evaluation, the protocol included high-resolution 3D structural sequences—T1-weighted, T2-weighted, and FLAIR. In pediatric patients, T2-weighted images were preferred over FLAIR due to superior gray-white matter contrast and reduced sensitivity to age-related myelination effects that can obscure lesion visibility on FLAIR. Additional sequences such as DWI and SWI were also acquired to provide a comprehensive diagnostic dataset. For subsequent algorithmic analysis, only the high-resolution T1-weighted data were used. Detailed acquisition parameters for the T1-weighted sequences, including TR, TE, flip angle, voxel size, and field of view, are summarized in Table 3. These parameters varied slightly depending on the scanner used—either Philips Achieva 1.5T (Motol University Hospital) or Philips Achieva 3 T (JL Clinic, Prague)—but were consistent with advanced clinical imaging standards for epilepsy surgery candidates. Healthy adult controls were examined using a Siemens Magnetom Vida 3 T scanner (Erlangen, Germany) with a standard 64-channel head coil. 3D MPRAGE T1-weighted data with submillimeter resolution were acquired (Table 4). For healthy pediatric controls full resolution 3D TFE T1-weighted data were acquired (Table 4). Both control groups were examined at MUH.

**Table 2:** Healthy subject groups and MRI parameters of T1-weighted sequences.

Group/Template	Number of subjects	MRI	TR [ms]	TE [ms]	Flip Angle	Voxel size [mm]	FOV [mm]
Adult subjects	81	3T	2 400	2.3	8	0.75 × 0.84 × 0.84	216 × 216
Pediatric subjects	36	1.5T	25	4.6	30	1 × 0.5 × 0.5	240 × 240

### MRI data processing

To streamline the processing workflow, all imaging datasets were converted from DICOM (Digital Imaging and Communication) format to NIfTI (Neuroimaging Informatics Technology Initiative) format using a MATLAB (MathWorks, USA) dcm2nii script, ensuring compatibility with batch processing on computing stations and serving as the primary input for each fully automatic detection algorithm.

### Normative template construction

For each modality—cortical thickness, gray matter extension, and gray–white matter junction—separate normative templates were constructed using data from pediatric (*N* = 36) and adult (*N* = 81) healthy control subjects, as described earlier. T1-weighted images were preprocessed through segmentation, spatial normalization, and smoothing. Subsequently, voxel-wise mean and standard deviation maps were generated across each control group to form modality-specific normative reference maps. These templates provided a statistical baseline against which patient data were compared. Individual z-score maps were then computed for each patient, enabling voxel-wise quantification of deviations relative to either age-matched or cross-age normative populations.

### Automatic detection of post-resection cavity

A MATLAB script was used to detect the post-resection cavity (PRC) mask. Pre- and post-resection data were read in NIfTI format and coregistered. Preoperative MRI was used as reference volume. The mean intensity value was calculated for each volume and the volumes were divided by this constant. This step normalized the intensities in the volumes. The next step was to convolute the data with a Gaussian kernel of 6 mm for smoothing. Subsequently, the post-resection data were subtracted from the pre-operative data. In the subtracted volume, voxels with positive value represent the locations where the difference between the two input volumes was found. A list of all such clusters was generated. Only the cluster corresponding to the post-resection cavity was retained. From the resulting binary mask, the volume of the post-resection cavity was calculated. PRC mask was taken as the gold standard for the further statistical processing (Fig. 3).

### FCD detection algorithms: cortical thickness, extension, and junction images

Fully automatic algorithms for detecting FCD were developed and implemented in MATLAB (2024a) using the Image Processing Toolbox and the SPM12 (version 7771) add-on toolbox (Fig. 4). These algorithms were designed to analyze T1-weighted MRI data and generate parametric maps based on cortical thickness, gray matter voxel intensities (extension images)^25^ and Grey Matter – White Matter (GM–WM) transitions (junction images)^26^. The resulting maps were compared to normative templates derived from control datasets, enabling the identification of candidate regions indicative of FCD. Our implementation was independently developed and is freely available for academic use. Unlike some other toolkits, which may require registration, licensing fees, or have restricted access policies, our pipeline can be shared without additional administrative or financial barriers, aside from the standard MATLAB license.

**Fig. 1:**
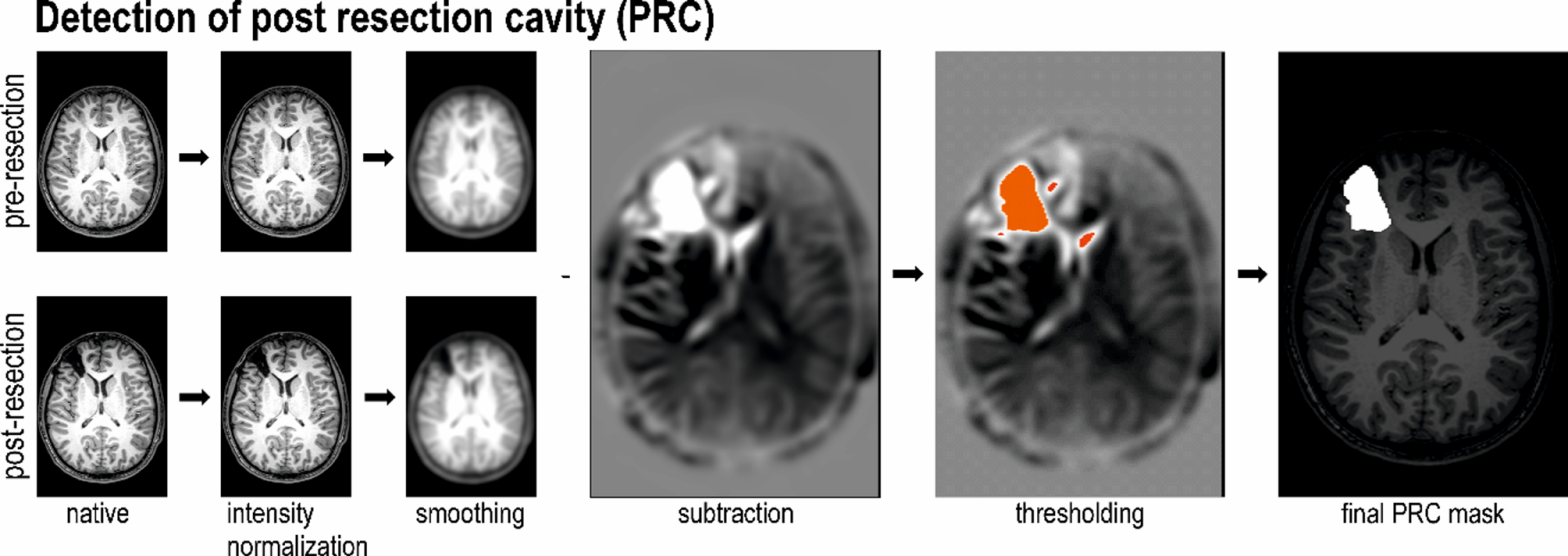
Detection of post resection cavity (PRC).

The detection algorithm based on cortical thickness utilized Freesurfer (version 7.0.2) and its “recon-all” pipeline to process all T1-weighted volumes for complete cortical reconstruction. The pipeline included motion and intensity corrections, transformation to Talairach space, white matter segmentation, and the calculation of cortical parameters. The cortex was parcellated into anatomical regions based on the Desikan-Killiany atlas^27^ and surface properties such as cortical width and curvature were computed. Statistical comparisons of cortical width between patients and healthy controls were performed using the “QDEC” function with a General Linear Model (GLM). The mri_glmfit script was employed to create design matrices, estimate parameters, and test for significant differences in cortical widths. The DODS (different offset, different slope) model was applied to assess deviations between groups. For each patient, a z-score map was generated and thresholded (z > 1.96, *p* < 0.05) to identify voxels or clusters with altered cortical widths.

For the detection algorithm based on extension images, abnormalities in gray matter voxel intensities were highlighted^25^. T1-weighted volumes were segmented into GM, WM, and cerebrospinal fluid with intensity normalization to correct inhomogeneities. The segmented volumes were normalized to the MNI standard template using affine and nonlinear transformations, and non-cortical regions such as the basal ganglia, brainstem, and cerebellum were excluded using a predefined mask. GM images were then smoothed with a three-dimensional Gaussian kernel (6 mm). Using data from the pediatric and adult control groups, templates were created by averaging the processed output volumes and calculating the mean and standard deviation for each voxel. Patient data were compared to these templates by simple subtraction with a predefined threshold or through the generation and.

**Fig. 2:**
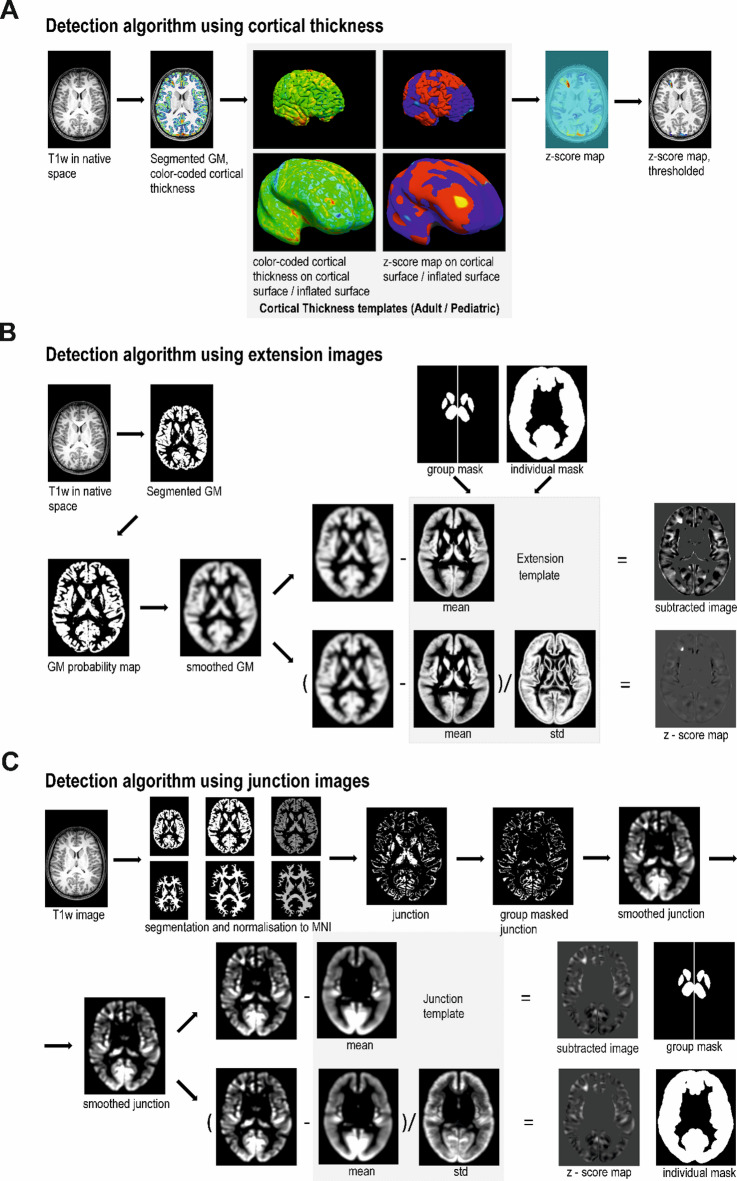
Overview of the processing pipelines used for automated lesion detection. The 3D surface renderings (pial surface and inflated brain with cortical thickness and z-score overlays) were generated using FreeSurfer software (version 7.2.0) and its built-in visualization tool Freeview (version 3.0) https://surfer.nmr.mgh.harvard.edu/. Other anatomical images were visualized using MRIcroGL https://www.nitrc.org/projects/mricrogl.

thresholding of z-score maps. Bright regions in the resulting extension images corresponded to intensity abnormalities in the cortex, with thresholding producing candidate clusters for FCD detection.

The detection algorithm based on junction images targeted areas with poorly defined gray-white matter transitions^26^. Similar to the extension images, T1-weighted volumes were segmented into GM, WM, and CSF, normalized to the MNI template, and non-cortical regions were excluded using the predefined mask. Mean and standard deviation values for GM and WM were calculated to define thresholds for converting the normalized T1-weighted volume into a binary image. Voxels with intensity values between the lower threshold (MeanGM + 0.5 × S.D.GM) and the upper threshold (MeanWM − 0.5 × S.D.WM) were assigned a value of 1, creating the binary image. This binary image was further smoothed using a three-dimensional Gaussian kernel (6 mm) to emphasize regions with clusters of blurred gray-white matter transitions. Templates for the pediatric and adult control groups were created by averaging the processed output volumes. Patient data were compared to these templates through subtraction and z-score mapping, where bright regions indicated cortical areas with poorly defined transitions.

Finally, the outputs from the cortical thickness, extension, and junction algorithms were integrated for a comprehensive analysis. Z-score maps (z > 1.96, *p* < 0.05) and subtracted volumes were thresholded to identify candidate regions for FCD. These analyses utilized both the Adult Subjects Template (AST) and the Pediatric Subjects Template (PST), providing robust comparisons across age groups and between patients and healthy controls.

### Neuroradiological MRI assessment

MRI data were reviewed by two experienced neuroradiologists: MK, with over 20 years of experience in neuroradiology, and ZH, with 5 years of experience, both specializing in epilepsy neuroimaging. High-resolution 3D T1-weighted sequences acquired on 1.5T or 3 T MRI scanners were included in the analysis, supplemented by 3D T2-weighted sequences for additional information. The MR images were acquired in the sagittal plane with multiplanar reconstructions in the axial and coronal planes. All image processing and analysis were performed using 3D Slicer software (slicer.org).

For the initial radiological assessment (first reading), the brain was divided into 11 predefined regions of interest (ROIs) per hemisphere, following the protocol described by Hulshof et al^28^. The ROIs included the frontal (medial, lateral, polar, basal, and central), temporal (medial and lateral), parietal (medial and lateral), and occipital (medial and lateral) regions. Each ROI was visually inspected for the presence of FCD and scored as definite, probable, or less likely based on radiological findings and individual expertise. Dysplastic features assessed included increased cortical thickness, blurring of the gray-white matter interface, transmantle sign, and signal abnormalities in the cortex and subcortical white matter.

Both radiologists independently evaluated the MRI data while blinded to all clinical information. The results were compared, and in cases of disagreement, the images were re-reviewed to achieve interobserver agreement. Discrepancies were resolved through discussion, resulting in either partial consensus (cases with remaining uncertainty) or full consensus. Lesions identified as visible were manually segmented by ZH, with MK providing agreement on lesion extent. These regions were marked as predictive radiological ROIs (PRRs).

The initial output of detection algorithms (Cortical Thickness, Extension, and Junction Images) was subsequently reviewed by the same two radiologists (second reading). Detected clusters were visually re-evaluated to determine whether they exhibited features characteristic of cortical widening or atypically blurred gray-white matter transitions. This comparative assessment allowed for the identification of clusters with a higher likelihood of false-positive detection. Areas exhibiting a high frequency of false positives encompass the amygdala, hippocampus, orbitofrontal cortex, and the region surrounding the anterior commissure. Based on these evaluations, a final group exclusion mask was developed to refine the algorithmic outputs. This exclusion mask was applied in the final analysis using all three detection algorithms to improve specificity and reduce false-positive rates.

It should be emphasized that PRRs represent purely radiological findings based on visible abnormalities identified by expert neuroradiologists on T1-weighted and T2-weighted (and/or FLAIR) sequences. In contrast, PRCs correspond to resected tissue as defined by multidisciplinary consensus, integrating data from multimodal imaging (MRI, PET, SPECT), stereo-EEG, and clinical evaluation. Resection cavities may extend beyond radiologically apparent lesions due to surgical strategy, anatomical constraints, or safety margins.

### ROC curves and areas under curves, comparison between algorithms

The performance of detection algorithms was evaluated using Receiver Operating Characteristic (ROC) curves, which provide a graphical representation of the trade-off between sensitivity and specificity at various threshold levels. All calculations were performed in MATLAB software using standard procedures^29^. The threshold values were set as the minimum and maximum value in the output grayscale image of the algorithm. For the selected values from this interval, the overlap rate with the resection cavity mask was determined (as True positive rate). The False positive rate was also measured for thresholded voxels that do not overlap with the resection cavity mask. True positive rate was calculated as the proportion of correctly detected voxels within the PRC mask relative to the total PRC volume. False positive rate was calculated as the proportion of detected voxels outside the PRC mask relative to the total brain volume excluding the PRC. This approach ensured that only voxels outside the PRC were used to compute false positive rates. The area under the curve (AUC) of ROC curve was used as a measure of overall accuracy.

### DICE score

The DICE similarity coefficient^30^ was calculated to evaluate the performance of the detection algorithms and manual radiological detection compared to the ground-truth, defined as the PRC mask. For each patient, the overlap between the detected regions (algorithm-based with threshold set to z-score > 2 or manually outlined) and the resection mask was computed. The DICE score reflects only the overlap between detected regions and the PRC mask (or PRR where applicable); voxels outside the ground-truth mask were not included in the calculation. False positive detections were instead considered in the ROC analysis via the false positive rate. The DICE score, a measure of spatial agreement, was calculated using own script in MATLAB. This metric quantifies the degree of correspondence, with a score of 1 indicating perfect match of detected clusters with PRR and 0 indicating no overlap. Higher DICE scores reflect better concordance between the detected regions and the ground-truth. As the PRCs are typically larger than the radiological lesions and often extend into structurally normal-appearing areas (e.g., white matter), only partial spatial overlap with algorithm-detected cortical clusters can be expected. Therefore, DICE scores are expcted inherently low and should not be interpreted as poor localization accuracy.

### Statistics and data presentation

Two-sample and paired-sample t-tests were applied as appropriate and conducted using MATLAB software, specifically the Statistics and Machine Learning Toolbox. For multiple comparisons, repeated-measures ANOVA with Holm-Šidák post hoc testing was performed using GraphPad Prism software. The Holm-Šidák correction was selected over more conservative Bonferroni correction as it provides greater statistical power in the context of moderately correlated tests. The level of statistical significance was set at 5%.

**Table 3:** Results of first radiological reading.

Subject	Two radiologists consensus	Confidence of radiological finding	localization	PRR volume [mm3]	PRC volume [mm3]	Overlay of PRR and PRC [mm3]	Percentual overlay of PRC with PRR
Subj01	Full	-	-	-	14769	-	-
Subj02	Full	Definite	F central sin	3466	52345	2741	5.2 %
Subj03	Full	Probable	T med sin	1164	21046	137	0.7 %
Subj04	Partial	Less likely	P lat sin	863	13798	0	0.0 %
Subj05	Partial	Definite	F lat sin	781	14029	685	4.9 %
Subj06	Partial	Probable	T lat sin	3691	26929	2902	10.8 %
Subj07	Partial	Probable	F lat sin	847	7920	127	1.6 %
Subj08	Partial	-	-	-	4287	-	-
Subj09	Full	Definite	F central dx	4701	3124	0	0.0 %
Subj10	Partial	Definite	P lat sin, O lat sin	1072	6274	453	7.2 %
Subj11	Full	-	-	-	6365	-	-
Subj12	Full	Definite	F polar dx	2558	18720	2211	11.8 %
Subj13	Full	-	-	-	9450	-	-
Subj14	Partial	-	-	-	14781	-	-
Subj15	Full	Definite	F lat dx	1217	4286	0	0.0 %
Subj16	Full	Definite	F med sin	3202	5999	745	12.4 %
Subj17	Partial	Probable	O lat dx	727	9432	447	4.7 %
Subj18	Full	Definite	F med sin	574	9690	449	4.6 %
Subj19	Partial	Definite	F polar dx	1593	3096	1056	34.1 %
Subj20	Full	-	-	-	12114	-	-
Subj21	Full	Probable	F med sin	1901	5307	351	6.6 %
Subj22	Full	Definite	F central dx	866	2385	0	0.0 %
Subj23	Partial	Less likely	F lat dx	1053	52716	0	0.0 %

## Results

During the initial radiological reading, full consensus was achieved in 13 out of 23 subjects (56%), indicating agreement on the presence or characteristics of these lesions. In 6 subjects (Subj01, 08, 11, 13, 14, and 20), no visible dysplastic features were identified. In all cases where lesions were not detected radiologically, patients underwent resection based on resection plans developed by an experienced MDT team. These plans incorporated multimodal data into the preoperative assessment, including the use of stereoEEG electrodes and PET imaging. Histological examination later confirmed the presence of an FCD IIB lesion in all cases except one (Subj11), where an FCD IIA lesion was identified.

A Predictive Radiological ROI (PRR) was manually plotted in 17 subjects, with an average volume of 1,781 mm³ range from 574 mm³ to 4,701 mm³ (see Table 1; Fig. 1). Automated post-resection cavity (PRC) detection successfully identified masks in all subjects, with an average volume of 13,863 mm³ range from 2,385 mm³ to 52,716 mm³. Overlap between PRR and PRC was observed in 12 subjects, with overlap percentages ranging from 0.7 to 34% and average DICE score 0,15 ± 0,03. No overlap was detected in five subjects (Table 1). In the 6 cases where radiologists were unable to identify epileptogenic lesion and to plot a PRR during the initial reading, the volume of the PRC did not significantly differ from those with MR-visible lesions (*p* = 0.4707). Since the PRC was successfully detected and incorporates the results of other modalities as well as the expertise of the multidisciplinary team, it was used as the ground-truth for evaluating detection performance.

All algorithm-based detection methods successfully identified potential FCD regions as positive clusters in all patients (three examples in Fig. 1). We quantified both the number of clusters overlapping with the PRC and their total volume. Detailed results for all subjects are presented in Table 2. To further evaluate algorithm performance, we calculated DICE scores of clusters detected from z-maps with a z-score threshold of 2 to quantify the spatial overlap between detected clusters and the PRC.

The median DICE scores (with interquartile range) were as follows: extension_adult 0.006 (0.018), extension_pediatric 0.0018 (0.010), junction_adult 0.028 (0.038), junction_pediatric 0.025 (0.026), cortical thickness_adult 0.006 (0.020), and cortical thickness_pediatric 0 (0). Among the tested algorithms, the junction algorithm demonstrated the best performance, as reflected by the greatest overlap with the PRC. Notably, the detection algorithms successfully identified FCD regions in MRI-negative cases, achieving DICE scores that did not significantly differ from those observed in MRI-positive patients (*p* = 0.444). As discussed later, these relatively low DICE scores are not necessarily indicative of poor localization, but rather reflect expected differences in volume and definition between algorithm-detected clusters and surgically resected tissue. The detected regions were typically smaller and more spatially constrained, often confined to cortical ribbon, while resections extended into surrounding areas including white matter or structurally normal cortex.

**Fig. 3:**
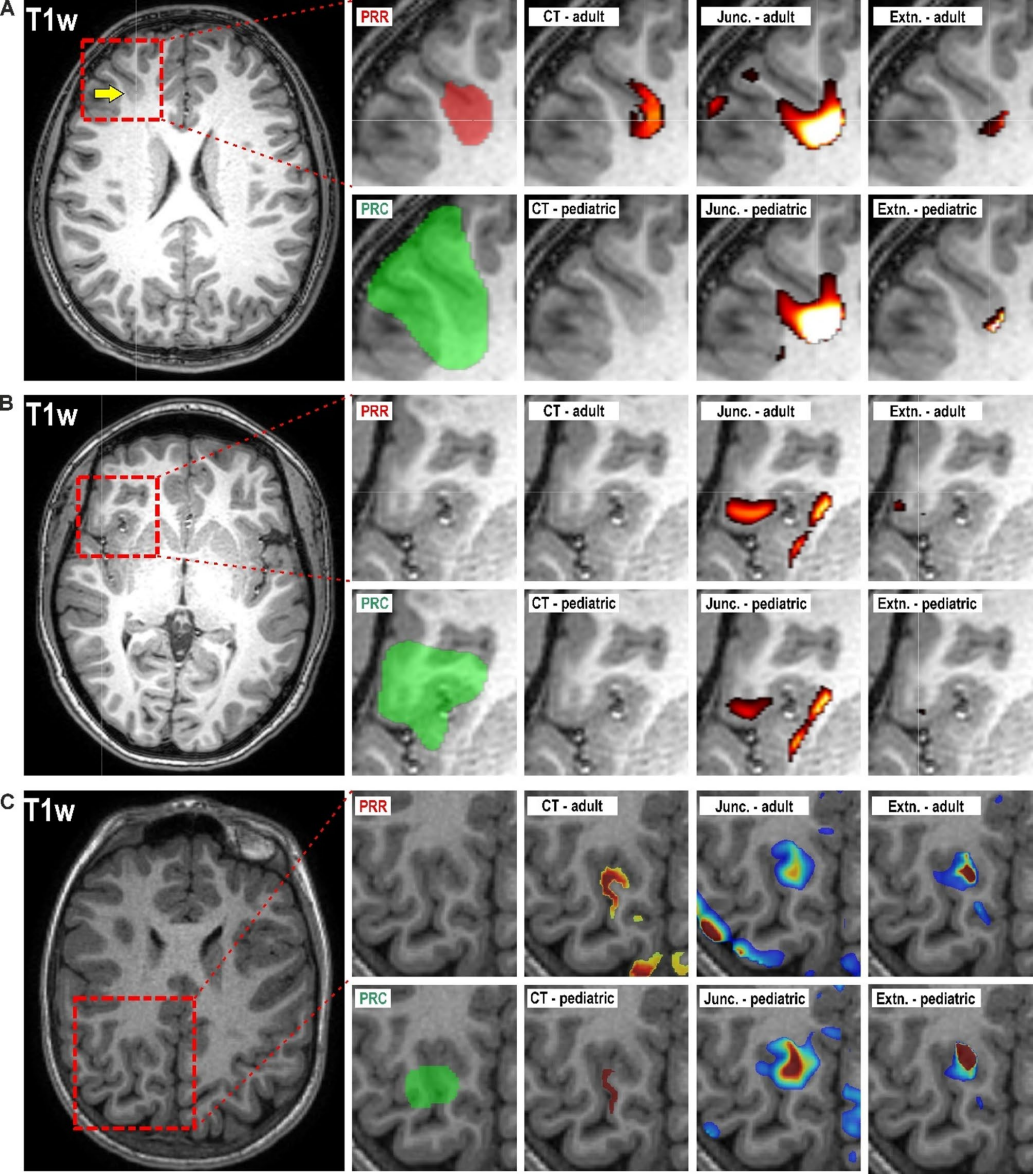
This figure presents three cases of pediatric FCD type II. **A -** a patient (Subject 12) with visible lesion successfully outlined by radiologist (PRR – red colored ROI). **B –** a patient (Subject 13) without visible lesion. **C** – (Subject 22) where two radiologists in consensus marked FCD tissue, however, final surgery was performed at different location and therefore PRR did not spatially correlate with PRC. Post resection cavity (PRC – green colored ROI, outputs of individual detection algorithms for both templates (detection threshold set to z-score > 2).

**Table 4:** Performance of detection algorithms – ROC curves. panel **A** – comparison to PRC, panel **B** – comparison to PRR.

	Cortical Thickness				Junction				Extension			
	Adult template		Pediatric template		Adult template		Pediatric template		Adult template		Pediatric template	
Subject	Clusters (PRC/Total)	% Overlay with PRC	Clusters (PRC/Total)	% Overlay with PRC	Clusters (PRC/Total)	% Overlay with PRC	Clusters (PRC/Total)	% Overlay with PRC	Clusters (PRC/Total)	% Overlay with PRC	Clusters (PRC/Total)	% Overlay with PRC
Subj01	0/9	0.0%	0/1	0.0%	4/67	16.6%	2/43	8.1%	1/11	0.0%	0/5	0.0%
Subj02	6/28	4.5%	0/3	0.0%	7/74	18.8%	5/64	6.3%	0/12	0.0%	0/4	0.0%
Subj03	3/17	1.7%	0/1	0.0%	6/89	29.4%	9/28	18.7%	2/12	0.6%	0/5	0.0%
Subj04	3/27	8.1%	3/4	1.5%	8/89	10.2%	8/77	3.0%	2/15	0.0%	1/6	0.7%
Subj05	2/48	0.5%	0/4	0.0%	4/105	8.5%	5/75	7.3%	1/15	1.6%	0/3	0.0%
Subj06	0/6	0.0%	0/0	0.0%	4/78	25.7%	1/39	20.2%	2/8	1.9%	1/6	0.4%
Subj07	0/19	0.0%	0/0	0.0%	3/124	4.2%	6/69	2.9%	0/11	0.0%	0/2	0.0%
Subj08	5/31	8.8%	0/18	0.0%	1/91	43.3%	1/48	46.7%	1/13	1.8%	0/6	0.0%
Subj09	1/19	0.8%	0/1	0.0%	2/117	2.2%	3/54	3.5%	0/10	0.0%	0/3	0.0%
Subj10	1/32	43.2%	1/10	20.7%	9/81	20.2%	10/67	22.3%	0/10	0.0%	0/4	0.0%
Subj11	1/12	1.6%	0/0	0.0%	2/94	25.9%	2/54	10.2%	0/12	0.0%	0/3	0.0%
Subj12	1/4	6.2%	0/0	0.0%	6/111	15.4%	3/73	9.2%	1/11	1.7%	1/3	1.3%
Subj13	0/20	0.0%	0/2	0.0%	3/77	7.0%	3/60	3.8%	0/8	0.0%	0/4	0.0%
Subj14	1/7	2.4%	1/1	0.6%	7/137	7.9%	7/78	6.1%	1/13	0.0%	1/4	0.0%
Subj15	0/6	0.0%	0/1	0.0%	5/100	10.3%	2/64	0.6%	0/3	0.0%	0/0	0.0%
Subj16	0/4	0.0%	0/0	0.0%	3/122	18.1%	3/53	13.2%	1/6	0.2%	2/5	0.1%
Subj17	0/8	0.0%	0/0	0.0%	3/76	6.4%	4/82	9.4%	0/8	0.0%	0/7	0.0%
Subj18	0/4	0.0%	0/0	0.0%	1/86	5.1%	3/52	7.1%	1/12	0.8%	1/8	1.0%
Subj19	1/9	0.1%	0/1	0.0%	3/52	29.2%	2/117	12.9%	0/6	0.0%	0/2	0.0%
Subj20	1/34	2.3%	0/6	0.0%	6/37	19.0%	10/67	14.4%	0/17	0.0%	2/2	0.0%
Subj21	5/9	0.8%	0/2	0.0%	2/108	16.2%	2/69	19.2%	0/5	0.0%	0/3	0.0%
Subj22	1/32	16.4%	1/11	10.6%	2/46	12.5%	2/83	26.2%	1/50	7.7%	1/5	6.3%
Subj23	1/11	0.3%	0/1	0.0%	7/80	25.7%	8/70	20.7%	3/11	0.7%	3/10	0.6%

We also compared algorithm performance between adult and pediatric templates. The junction algorithm showed significantly higher performance with the adult template compared to the pediatric template (*p* = 0.022). The other algorithms did not show significant differences individually. However, when considering all algorithms collectively, the adult template significantly outperformed the pediatric template (*p* < 0.001).

To evaluate how detection results varied over range of all thresholds, we reconstructed ROC curves and analyzed their AUC for all algorithms and both templates. Two independent comparisons were performed using different ground-truths. First, the results were compared to the PRC, which is typically larger than the PRR due to the multimodal data and surgical planning strategy. No significant differences were observed between the Adult and Pediatric templates across all detection pipelines. Similar findings were obtained when comparing the results to the PRR. However, higher AUC values were observed for ROC curves constructed using the PRR (0.65 ± 0.08) compared to those based on the PRC (0.56 ± 0.04) (Fig. 2). All AUC and 95% confidence intervals are summarized in Supplementary Table S1.

**Fig. 4:**
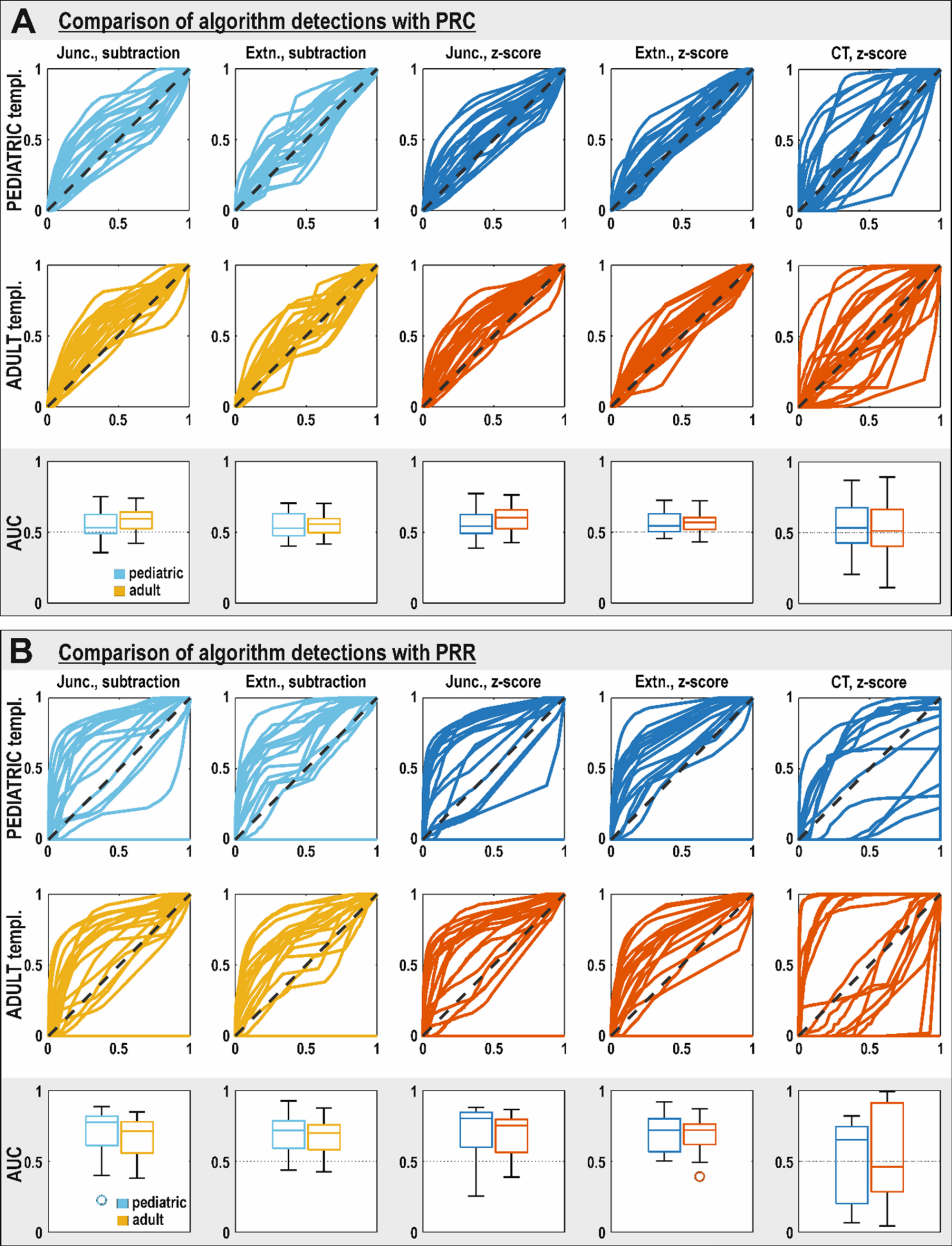
ROC curves for different algorithms and used templates. **A** - comparison with PRC, **B** - comparison with PRR. Boxplots: AUC comparison between ADULT and PEDIATRIC template – colors match with ROC curves. (Junc – junction, Extn. – extension, CT – cortical thickness). AUC values of ROC curves are unitless.

## Discussion

In this study, we evaluated the performance of radiological assessment and automated detection algorithms for identifying FCD type II lesions in pediatric epilepsy patients. Algorithms targeting cortical blurring and cortical thickness abnormalities were applied using both adult and pediatric templates and validated against two ground truths: the post-resection cavity (PRC) and predictive radiological ROIs (PRR). Among the methods, the junction algorithm consistently demonstrated the highest detection accuracy, particularly when paired with the adult template. Template selection significantly influenced performance, with all algorithms performing better when the adult normative template was used, highlighting the importance of age-consistent but developmentally stable references in pediatric imaging.

### Automated detection in MRI-negative FCD II: enhancing surgical planning

Notably, the algorithms identified positive clusters within the PRC even in patients whose MRI scans were deemed negative by experienced neuroradiologists. Improving lesion detection in such MRI-negative FCD type II cases is critical, as early identification of surgical candidates can significantly enhance treatment outcomes. Despite typical radiological features like gray–white matter blurring and cortical thickening, a substantial proportion of FCD II lesions remain undetected on conventional MRI. Previous studies have reported MRI-negative rates ranging from 15 to 40% in FCD II patients, underscoring the diagnostic challenge and its association with poorer surgical outcomes^31–34^.

In our cohort, six patients were classified as non-lesional based on visual assessment, despite having undergone high-resolution imaging. Interestingly, five of these patients had histologically confirmed FCD IIB and one had FCD IIA. While IIB lesions are generally more conspicuous than IIA, their features were insufficiently distinct to meet consensus criteria for radiological detection. Lesions were marked only when typical signs, such as cortical thickening, junctional blurring, the transmantle sign, or subcortical signal changes, were clearly expressed. Moreover, neuroradiological evaluation was conducted without clinical or multimodal imaging data, which likely contributed to lesion invisibility. These findings illustrate the inherent limitations of standard MRI interpretation and support the need for advanced, algorithm-driven approaches to detect subtle abnormalities and guide surgical decision-making.

### Radiological predictions vs. surgical resection: interpreting DICE scores

The spatial overlap between radiological predictions (PRR) and surgical resections (PRC) was relatively low, with an average DICE score of 0.15 ± 0.03. This modest agreement is expected, given the conceptual and anatomical differences between these two reference standards. PRRs represent MRI-visible abnormalities identified by neuroradiologists, while PRCs reflect the extent of resected tissue defined by a multidisciplinary team, integrating multimodal data (e.g., SEEG, PET, SPECT, functional mapping) and accommodating the need for surgical access.

Importantly, PRCs are typically larger than the radiological lesion and often extend into subcortical white matter or adjacent areas not directly abnormal on MRI. In contrast, algorithm-detected clusters, especially after statistical thresholding, are spatially restricted and typically confined to small cortical regions. Given this volume asymmetry, low DICE scores are an expected outcome and do not reflect poor localization accuracy. Rather, they highlight the inherent mismatch between focal detection outputs and the broader anatomical targets required for effective resection of epileptogenic zone.

### Improving detection of subtle FCD II lesions

Our study aligns with previous research highlighting the potential of algorithm-based methods to improve FCD detection, particularly for type II lesions^25,26^. Advanced algorithms, such as those designed to detect cortical blurring, have demonstrated significant improvements in sensitivity, especially in challenging MRI-negative cases, as noted in prior studies^13,35^. These findings are consistent with reports from the Multi-centre Epilepsy Lesion Detection (MELD) project group, which achieved an 85% sensitivity for FCD detection using a surface-based machine learning algorithm, particularly for type II lesions. The MELD study also emphasized the ability of advanced methods to integrate multicenter data, enhancing detection sensitivity even in heterogeneous imaging environments^16^. Similarly, our results underscore the potential of automated detection algorithms to address the limitations of visual interpretation, particularly in cases where radiological assessment fails to identify subtle cortical abnormalities.

### Effect of template selection on algorithm performance in pediatric FCD

The performance of FCD detection algorithms is heavily influenced by the templates used. Previous studies, such as those by David et al.^36^ and Spitzer et al.^16^ have shown that larger and more consistent templates improve classification performance and algorithm generalizability. In our study, algorithms performed significantly better with the Adult template than the Pediatric template. This difference likely reflects greater anatomical consistency in the adult population, where brain structures are fully developed and relatively stable. In contrast, the pediatric template was derived from a smaller and more heterogeneous group, where ongoing brain maturation introduces substantial variability in cortical morphology. Such developmental changes can reduce the effectiveness of normalization and compromise the sensitivity of detection algorithms. Division of pediatric templates into narrower age subgroups could reduce variability and improve detection accuracy. This aligns with Adler et al.^20^who highlighted that algorithms based on adult populations often fail to generalize well to pediatric patients because of differences in disease presentation and brain development stages. These anatomical and pathological distinctions frequently result in lower sensitivity and specificity in pediatric settings. Similarly, Dangouloff-Ros et al.^37^ emphasized the need for pediatric-specific algorithms and templates, as age-specific designs better capture developmental nuances. This is further supported by Demerath et al.^38^who demonstrated the critical role of consistent imaging protocols and refined templates in optimizing detection outcomes.

### Comparative performance of detection algorithms in pediatric FCD

The junction algorithm, focusing on detecting blurring of the gray-white matter interface, demonstrated superior performance in identifying FCD type II lesions in our study of pediatric population. This aligns with previous findings by Jin et al.^35^ and Adler et al.^20^which emphasized the sensitivity of junctional features in capturing subtle morphological changes associated with FCD. Unlike cortical thickness-based methods, which are influenced by developmental variations in cortical thickness across age groups^39^junctional features appear less dependent on age-specific changes, providing more robust detection in pediatric cohorts where brain morphology is highly variable.

### Controlling false positives in automated FCD detection

A persistent challenge in automated FCD detection is the high rate of false positive clusters, particularly in areas of normal anatomical variability such as the insula, cingulate gyrus, and medial occipital cortex. These regions often exhibit features, such as natural blurring or cortical thickness variation, that can mimic FCD in algorithmic outputs. To address this issue, we implemented an anatomical exclusion mask derived from control subjects to suppress clusters consistently occurring in non-pathological regions. This strategy improved specificity by suppressing non-pathological detections observed in healthy brains.

However, this approach may also reduce sensitivity if true lesions are located within the excluded areas. While this trade-off helps reduce overdiagnosis, future work should aim to refine exclusion strategies using larger normative datasets or probabilistic anatomical atlases, enabling better preservation of lesion detectability in high-risk regions. Future generations of algorithms could use probabilistic lesion maps or ensemble classifiers to reduce false positivity in these regions without compromising sensitivity.

To assess false positive behavior, we also applied the detection algorithms to healthy controls. While small, spatially scattered clusters were occasionally observed, these were typically located near anatomical boundaries and had z-scores just above the threshold (z > 2). Crucially, without a radiological ground truth in controls, these findings cannot be directly quantified. However, their fragmented appearance and low z-scores support the clinical recommendation to interpret full z-score maps rather than relying solely on thresholded outputs. This approach helps distinguish meaningful detections from noise, especially in subtle or MRI-negative cases.

### Clinical value of sub-threshold clusters

In this study, DICE scores were calculated from clusters detected after applying a Z-score threshold of > 2, which is a conventional approach to select statistically significant results. While this provides a rigorous quantitative benchmark, it does not fully capture the clinical reality. In several cases, clusters below this statistical threshold still provided meaningful guidance to clinicians when interpreted alongside multimodal data such as stereo-EEG or PET findings. Importantly, these sub-threshold clusters—while not contributing to the formal DICE score—were sometimes located in regions ultimately identified as part of the epileptogenic zone. This phenomenon is illustrated in Supplementary Figure S1, where a sub-threshold cluster corresponded to the resected region in an MRI-negative patient. These observations suggest that, although statistical thresholding is valuable for performance reporting, clinical interpretation of algorithm outputs should remain flexible and context-driven, especially in MRI-negative or diagnostically challenging cases.

### Limitations of cortical thickness algorithms in FCD IIB lesions

In several patients (Subjects 16, 17, 21, and 22), ROC performance for the cortical thickness algorithm was unexpectedly poor, with curves falling below the diagonal. On visual inspection, these lesions often exhibited T1 hyperintensity within the cortex or an associated transmantle sign. These signal alterations likely disrupt tissue segmentation by reducing gray–white matter contrast, leading to artificial thinning or thickening of the cortical boundary and resulting in unreliable z-scores. Notably, such patterns was previously associated with balloon cells in FCD IIB, which have been reported to alter signal intensity and confound standard structural metrics^40^. These cases highlight a limitation of the cortical thickness algorithm in certain lesion subtypes, particularly those with abnormal myeloarchitecture or signal patterns, and suggest a need for caution when interpreting algorithm output in atypical presentations.

### Clinical integration and generalizability

Beyond diagnostic performance, a key strength of the proposed algorithmic framework lies in its potential for integration into clinical workflows. Unlike deep learning models, which often lack interpretability, our feature-based methods generate anatomically meaningful outputs that can be reviewed alongside conventional MRI. This facilitates clinical trust and integration with multimodal data. Moreover, rule-based methods are less prone to overfitting, making them well suited for small pediatric cohorts where training deep models are difficult to train reliably. As such, our approach offers a practical decision-support tools, particularly valuable for radiologists with limited experience in epilepsy imaging. The ability to detect clusters in MRI-negative cases further underscores their value in challenging diagnostic scenarios, potentially guiding additional investigations such as SEEG or PET. Notably, the algorithms demonstrated robust performance across a range of scanner vendors, field strengths (1.5T and 3 T), and acquisition protocols. This generalizability supports their potential for broader clinical adoption, even in heterogeneous imaging environments where more constrained AI models may struggle. While our approach is conceptually related to the Morphometric Analysis Program (MAP) framework originally proposed by Huppertz et al., all algorithms used in this study were implemented in-house and do not rely on the commercial MAP18 toolbox. This ensures that our methods are fully transparent, adaptable, and freely available for academic and clinical use.

### Limitations and future directions

Our study has several limitations. The relatively small sample size, typical for pediatric DRE cohorts, may limit generalizability. Notably, our dataset did not include patients under 2 years of age, which may limit applicability to this subgroup where incomplete myelination can affect lesion visibility on T1-weighted MRI. However, focusing on this underrepresented population provides critical insights into detection challenges exacerbated by developmental variability in brain morphology. The retrospective design introduces potential bias, as all patients were epilepsy surgery candidates, potentially favoring more severe cases. To address this, we utilized two ground truths: PRR, derived from expert radiological assessment, and PRC, based on surgical outcomes. PRC allowed validation of algorithms in MRI-negative cases, highlighting their ability to detect clusters missed by radiologists. However, as PRC reflects multimodal data and surgical planning, it may not always align with the true epileptogenic zone or structural lesion. Despite this, algorithms consistently detected clusters within PRC, demonstrating their potential to complement visual evaluation.

A further limitation of this study lies in the heterogeneity of the pediatric template, which reflects the considerable anatomical variability across developmental stages. This reinforces the need for age-stratified templates to better account for brain maturation processes and improve algorithm performance in younger pediatric subgroups. To enhance template quality and confirm the generalizability of detection algorithms, future prospective multi-center studies should include larger, age-stratified pediatric cohorts, especially focusing on infants under two years of age, whose ongoing myelination affects brain morphology and presents specific imaging challenges.

## Conclusion

In conclusion, this study highlights the potential of detection algorithms to improve the diagnostic accuracy of FCD type II in pediatric patients. Among the tested methods, the junction algorithm, focusing on gray-white matter blurring, demonstrated the best performance. Template choice was also important factor, with the adult template yielding superior performance compared to the pediatric template. Notably, the use of both PRR and PRC as ground truths provided a robust framework for evaluating algorithmic performance, enabling the validation of clusters even in MRI-negative cases. While the PRC reflects multimodal data and surgical strategy, which may not perfectly match the true epileptogenic zone, it offered a valuable benchmark for algorithmic detection in challenging cases. This dual-ground-truth approach also facilitated comparisons with radiologists, highlighting the potential for algorithms to assist less experienced practitioners in detecting subtle FCD lesions. Future studies should explore larger, prospectively collected cohorts and refined templates to optimize algorithm performance.

## Supplementary Information

Below is the link to the electronic supplementary material.

Supplementary Material 1

## Data Availability

The primary clinical data analyzed in this study are not publicly available due to limitations in the ethical approval granted by the institutional review board. Access to the data may be considered upon reasonable request to the corresponding authors, subject to appropriate ethical and legal restrictions. All scripts and custom functions developed for this study are publicly available on GitHub at https://github.com/Jasan-CZ/FCD_detection_algorithms.

## Code availability

The image processing and lesion detection pipelines (cortical thickness, extension, and junction algorithms) were implemented in MATLAB R2024a using SPM12 (version 7771) and Freesurfer (version 7.2.0). All scripts and custom functions developed for this study are publicly available on GitHub at https://github.com/Jasan-CZ/FCD_detection_algorithms. The pipeline is compatible with standard T1-weighted MRI data and integrates with widely used open-source neuroimaging tools. Additional documentation or support for implementation may be provided by the authors upon reasonable request.
