# Supplementary material for: A comparative analysis of imaging-based algorithms for detecting focal cortical dysplasia type II in children
Source: Sci Rep. 2025 Aug 15;15:29946. doi: 10.1038/s41598-025-16015-3 (PMC12356853; doi:10.1038/s41598-025-16015-3)

Title: A Comparative Analysis of Imaging-Based Algorithms for Detecting Focal Cortical Dysplasia Type II in Children

Authors: *Jan Šanda et al.*

Supplementary Table S1. Area Under the Curve for ROC curves. Mean AUC ± 95% CI

Ground Truth	Algorithm Template	junction_z-score		junction_subtraction		extension_z-score		extension_subtraction		CT_z-score	
		adult	pediatric	adult	pediatric	adult	pediatric	adult	pediatric	adult	pediatric
PRC		0.6±0.04	0.56±0.04	0.59±0.03	0.55±0.04	0.57±0.03	0.56±0.03	0.55±0.03	0.55±0.04	0.53±0.07	0.55±0.07
PRR		0.68±0.06	0.7±0.08	0.67±0.06	0.69±0.08	0.69±0.05	0.7±0.05	0.68±0.05	0.69±0.06	0.57±0.14	0.4±0.13

Supplementary Figure S1. Patients (Subject 23) that was classified as MRI negative. **A** - PRC shows resection that was planned based on stereo EEG and PET. **B** – result of Cortical Thickness algorithm without and with use of Zscore>2 threshold.

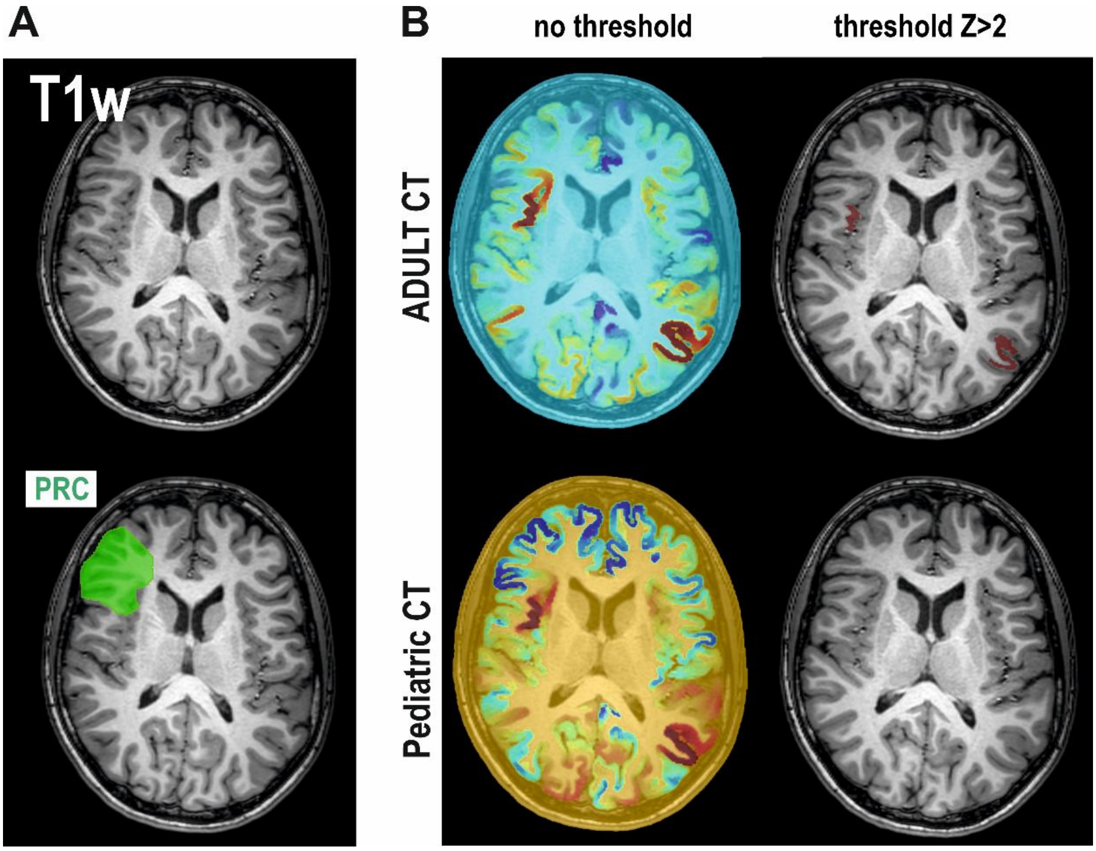

Supplement: Supplementary file 1 — Supplementary Material 1 [file 41598_2025_16015_MOESM1_ESM.pdf]
